# CYP24A1 Mutation in a Girl Infant with Idiopathic Infantile Hypercalcemia

**DOI:** 10.4274/jcrpe.4841

**Published:** 2018-02-26

**Authors:** Jens Otto Broby Madsen, Sabrina Sauer, Bodo Beck, Jesper Johannesen

**Affiliations:** 1 Herlev University Hospital, Department of Pediatrics, Herlev Ringvej, Herlev, Denmark; 2 University Hospital of Cologne, Institute of Human Genetics, Kerpenerstr, Cologne, Germany; 3 University of Copenhagen Faculty of Health and Medical Sciences, Blegdamsvej, København, Denmark

**Keywords:** Idiopathic infantile hypercalcemia, CYP24A1, nephrocalcinosis, vitamin-D supplementation

## Abstract

Idiopathic infantile hypercalcemia (IIH) was associated with vitamin-D supplementation in the 1950’s. Fifty years later, mutations in the CYP241A gene, involved in the degradation of vitamin-D, have been identified as being a part of the etiology. We report a case of a 21-month old girl, initially hospitalized due to excessive consumption of water and behavioral difficulties. Blood tests showed hypercalcemia and borderline high vitamin-D levels. Renal ultrasound revealed medullary nephrocalcinosis. An abnormality in vitamin-D metabolism was suspected and genetic testing was performed. This revealed the patient to be compound heterozygous for a common (p.E143del) and a novel (likely) disease-causing mutation (p.H83D) in the CYP24A1 gene. The hypercalcemia normalized following a calcium depleted diet and discontinuation of vitamin-D supplementation. Increased awareness of the typical symptoms of hypercalcemia, such as anorexia, polydipsia, vomiting and failure to thrive, is of utmost importance in diagnosing IHH early and preventing long-term complications such as nephrocalcinosis. Further identification of as many disease-causing mutations in the CYP24A1 gene as possible can help identification of predisposed individuals in whom vitamin-D supplementation should be reconsidered.

## What is already known on this topic?

In 2011 new research found that Idiopathic infantile hypercalcemia (IIH) was associated with mutations in the CYP24A1 gene involved in vitamin-D metabolism. CYP24A1 is responsible for the degradation of both 1,25-dihydroxyvitamin-D3 and the precursor 25-hydroxyvitamin-D3. Thus, reduced activity will increase the level of active vitamin-D and can lead to hypercalcemia. So far only a limited number of mutations have been reported.

## 

### What this study adds?

The patient presented here reveals a unique clinical presentation and a new mutation expanding the mutational spectrum of CYP24A1 associated IIH.

## Introduction

IIt took almost 50 years for idiopathic infantile hypercalcemia (IIH), from first identification to the discovery of its etiology by Schlingmann et al (1) who identified mutations in the *CYP24A1* gene in patients with IIH (1). *CYP24A1* is responsible for the degradation of oth 1,25-dihydroxyvitamin D_3_ and the precursor 25-hydroxyvitamin-D_3_. Thus, reduced activity will increase the level of active vitamin-D and can lead to hypercalcemia. So far only a limited number of mutations have been reported (2). The patient presented here reveals a unique clinical presentation and a new mutation, expanding the mutational spectrum of *CYP24A1* associated IIH.

## Case Report

A 21-month old girl was hospitalized in order to observe her excessive thirst and failure to thrive. For five months, the girl had been drinking extensively day and night and had been less interested in eating solid foods. Restriction of drinking made the girl refuse to eat altogether. Her general practitioner had ruled out diabetes mellitus by blood tests and diabetes insipidus by evaluating concentration of urine.

The patient’s history revealed a healthy pregnancy and birth. However, severe problems with vomiting occurred in the first seven months of life. The infant had been breastfed but the feedings had been supplemented with formula, as she always seemed hungry. After introduction of solid foods, the hunger and vomiting diminished. There was no family history of similar problems. There was no consanguinity: the mother was of Iranian and the father of Danish descent. The infant received no medication besides the recommended 400 IU/day of vitamin-D.

Physical examination was normal and there were no syndromic stigmata nor signs of physical disease. Her growth chart revealed normal height for age but her weight had decreased by one standard deviation within the three preceding months.

**Figure 1:**
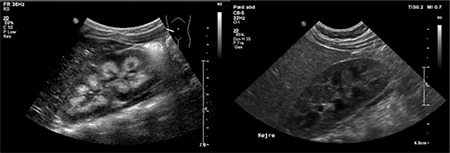
Ultrasound of the kidneys of the index patient and her brother. To the left, ultrasound of the index patient revealed kidney enlargement with hyperechogenic pyramids and signs of medullary nephrocalcinosis. To the right, ultrasound of the younger brother showed normal echogenicity of pyramids

When hospitalized, blood tests showed high levels of both total calcium (3.42 mmol/L; Ref. range 2.17-2.66 mmol/L) and free calcium (1.68 mmol/L; Ref. range 1.18-1.32 mmol/L). Parathyroid hormone (PTH) level was undetectable (<4 ng/L; Ref. range 14.0-72.0 ng/L). 25 vitamin-D and 1.25 vitamin-D levels were in the high-to-normal range. 25 vitamin-D level was 107 nmol/L (Ref. range >50 nmol/L), and similarly 1.25 vitamin-D level was 146 pmol/L (Ref. range 51-177 pmol/L). Serum phosphate level, liver, kidney and thyroid tests were all normal. Ultrasound of the kidneys revealed medullary nephrocalcinosis (Figure 1). Her urine was found to be hypercalciuric with a Ca/creatinine ratio of 1.9 mmoL/mmoL (Ref. range: <0.7 mmoL/mmoL). There were no signs of other diseases or malignancy. Both parents had blood calcium levels tested, and the mother had a calcium level of 2.53mmol/L (Ref. range: 2.15-2.51mmol/L) that was considered normal and no further examinations were initiated, including renal ultrasound or renal testing.

**Figure 2:**
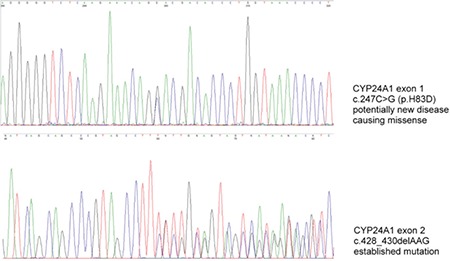
Electropherogram after genetic analysis of the index patient. Genetic investigation revealed the girl to be a compound heterozygote with two different mutations in the CYP24A1 gene: the previously reported c.428_430delAAG (p.E143del) mutation, and the novel c.247C>G (p.H83D) mutation

Genetic testing showed the girl to be compound heterozygous for the following CYP24A1 mutations: c.428_430delAAG (p.E143del) and c.247C>G (p.H83D) (Figure 2). Testing of the non-symptomatic parents revealed that the well-known p.E143del mutation was inherited from the heterozygotic mother and the novel p.H83D mutation was inherited from the heterozygotic father.

The patient was started on a low calcium diet and vitamin-D supplementation was stopped resulting in the calcium and PTH levels returning to normal within the subsequent five months. Since then the patient’s serum calcium level has remained within the acceptable range, while milk and other high calcium products have been slowly reintroduced. Informed consent was obtained from the family.

## Discussion

More than 20 different mutations affecting the *CYP24A1* gene have been reported (2). Of the 37 symptomatic cases recently published, 14 were adults (older than 18 years), three were between three and 13 years of age and the remainder were less than one year old at diagnosis. The average age at diagnosis (excluding the adults) was 11 months, ranging from one month to 13 years.

Symptoms such as polyuria, polydipsia, anorexia, fatigue and depression are all known to be associated with hypercalcemia, but are easily misinterpreted (3). In small children, failure to thrive is a common symptom of IIH and within the 23 pediatric cases, 74% were described as having weight loss and failure to thrive at presentation (1,4,5,6,7,8). In older children and adults urolithiasis can be the only symptom (6,8,9). Though all patients with symptoms had signs of nephrocalcinosis, no correlation was reported between type of mutation and clinical presentation (1,4,5,6,7,8,9).

Our patient showed several symptoms of hypercalcemia as an infant, but had no blood tests performed and was diagnosed as a case with simple regurgitation. Blood tests were performed when weight gain was affected. The finding of increased levels of calcium and high-to-normal 1,25(OH)D in the face of suppressed PTH levels, usually points to vitamin-D related disease and can be considered typical for IIH (1). There was no history of vitamin-D overdosing as our patient received the recommended supplementation of 400 IU/day. As there were no signs of William’s syndrome, familial hypercalcemia or other diseases, the hypercalcemia had earlier been described as idiopathic.

The well-known p.E143del mutation is clearly pathogenic (2), whereas the p.H83D missense variant is a novel finding in a patient with IIH. The fact that c.247C>G is a rare variant that cannot be found in any known genome variant databases (ExAC genome browser; Exome variant server, dbSNP), together with the typical recessive segregation pattern in the family with asymptomatic, heterozygous parents, all suggest biallelic loss of CYP24A1 function, caused by compound heterozygosity for p.E143del; p.H83D in our patient. This conclusion is further supported by the bioinformatic prediction performed with PolyPhen-2 and MutationTaster-2 (10,11). Both programs predict the p.H83D variant to be probably damaging and disease causing.

Dinour et al (6) described four heterozygous children all carrying p.E143del mutations. Even though none of them displayed symptoms of hypercalcemia, all of them had blood levels of calcium at the very upper end of the normal range. This is interesting since none of them received vitamin-D supplementation. This clearly indicates that the role of vitamin-D supplementation, in carriers of CYP24A1 mutations, is not fully understood. Further these cases supports the pathogenicity of the newfound mutation, since our patient had clearly elevated calcium levels. At birth, the younger brother of our patient was tested and found to be carrier of only the p.E143del mutation. At four months of age, blood tests revealed both elevated total calcium levels of 2.75 mmol/L (Ref. range: 2.10-2.62) and free calcium levels of 1.48 mmol/L (Ref. range: 0.18-1.32). However, 25(OH)D was low with a value of 31 nmol/L (Ref. range: >50 nmol/L). The urine was hypercalciuric with a Ca/creatinine ratio of 1.7 mmol/mmol (Ref. range: <0.7 mmol/mmol), but ultrasound revealed no signs of nephrocalcinosis (Figure 1). At 12 months of age the brother was still healthy and had normal urinary calcium excretion, but regular follow-up to observe for development of nephrocalcinosis continues.

Treatment of hypercalcemia has differed significantly in patients with IIH. Oral hydration and discontinuation of vitamin D supplementation was sufficient to normalize the calcium levels in our patient. However most of the reported patients have been treated with both iv hydration, furosemide, and in some severe cases with corticosteroids and bisphosphonates (1,3,4,5,6,7,8). One patient, who was found to be homozygous for the well-known R396W mutation, even had to undergo hemodialysis (4). Accordingly, the severity of hypercalcemia at diagnosis is not only related to the underlying mutation, but also dependent upon the time delay to diagnosis as well as vitamin-D supplementation (5).

Though treatment can often be limited to calcium restriction, calcium metabolism probably remains affected throughout life (5,6). In two publications, periodically high calcium levels, 11 and 18 years post diagnosis, were reported and in one case the nephrocalcinosis remained unchanged after 18 years (1,5).

Since CYP24A1 mutations clearly result in a genetic disposition to hypercalcemia, we recommend genetic testing in siblings of IIH patients, so that vitamin-D supplementation can be reconsidered. A similar conclusion was reached by Ammenti et al (12) after reviewing data on nephrocalcinosis (not solely caused by IIH), stating that early recognition was associated with catch-up growth and stabilization of glomerular function.

Further, all patients with mutations should undergo regular follow-up, since hypercalcemia and nephrocalcinosis have been demonstrated in an asymptomatic compound heterozygote boy, even though he was not receiving any vitamin-D supplementation (1). Certainly it is interesting to follow these predisposed patients to obtain data on long-term prognosis and specifically so in situations where vitamin-D supplementation is normally considered, such as in treatment of osteoporosis (9).
